# Targeting COVID-19 and Human Resources for Health News Information Extraction: Algorithm Development and Validation

**DOI:** 10.2196/55059

**Published:** 2024-10-30

**Authors:** Mathieu Ravaut, Ruochen Zhao, Duy Phung, Vicky Mengqi Qin, Dusan Milovanovic, Anita Pienkowska, Iva Bojic, Josip Car, Shafiq Joty

**Affiliations:** 1 Nanyang Technological University Singapore Singapore; 2 Episteme Systems Geneva Switzerland; 3 King's College London London United Kingdom; 4 Salesforce Research San Francisco, CA United States

**Keywords:** COVID-19, SARS-CoV-2, summary, summarize, news articles, deep learning, classification, summarization, machine learning, extract, extraction, news, media, NLP, natural language processing

## Abstract

**Background:**

Global pandemics like COVID-19 put a high amount of strain on health care systems and health workers worldwide. These crises generate a vast amount of news information published online across the globe. This extensive corpus of articles has the potential to provide valuable insights into the nature of ongoing events and guide interventions and policies. However, the sheer volume of information is beyond the capacity of human experts to process and analyze effectively.

**Objective:**

The aim of this study was to explore how natural language processing (NLP) can be leveraged to build a system that allows for quick analysis of a high volume of news articles. Along with this, the objective was to create a workflow comprising human-computer symbiosis to derive valuable insights to support health workforce strategic policy dialogue, advocacy, and decision-making.

**Methods:**

We conducted a review of open-source news coverage from January 2020 to June 2022 on COVID-19 and its impacts on the health workforce from the World Health Organization (WHO) Epidemic Intelligence from Open Sources (EIOS) by synergizing NLP models, including classification and extractive summarization, and human-generated analyses. Our DeepCovid system was trained on 2.8 million news articles in English from more than 3000 internet sources across hundreds of jurisdictions.

**Results:**

Rules-based classification with hand-designed rules narrowed the data set to 8508 articles with high relevancy confirmed in the human-led evaluation. DeepCovid’s automated information targeting component reached a very strong binary classification performance of 98.98 for the area under the receiver operating characteristic curve (ROC-AUC) and 47.21 for the area under the precision recall curve (PR-AUC). Its information extraction component attained good performance in automatic extractive summarization with a mean Recall-Oriented Understudy for Gisting Evaluation (ROUGE) score of 47.76. DeepCovid’s final summaries were used by human experts to write reports on the COVID-19 pandemic.

**Conclusions:**

It is feasible to synergize high-performing NLP models and human-generated analyses to benefit open-source health workforce intelligence. The DeepCovid approach can contribute to an agile and timely global view, providing complementary information to scientific literature.

## Introduction

The unprecedented outbreak and rapid spread of COVID-19 have led to detrimental impacts on almost the whole population worldwide. Early detection of such an outbreak or its impact on the population can help policymakers identify intervention points and set priorities and policies [1,2]. This detection, also called public health surveillance (PHS), is defined as “the continuous, systematic collection, analysis, and interpretation of health-related data needed for the planning, implementation, and evaluation of public health practice” [3,4]. Traditional PHS, which is mostly passively conducted, is often limited by data quality and timeliness, restricting the accurate and quick or even instantaneous identification of outbreaks and subsequent impacts and adoption of an effective intervention [2,5]. PHS has evolved over time as technological advances provide an opportunity for more accurate and timely information collection and analysis [6].

Data-driven artificial intelligence is one of the innovative technologies that can address the limitation of traditional PHS [7]. The open-source textual data from publicly available sources that is of high frequency, high volume, and relatively low effort to collect provide a great potential for the application of natural language processing (NLP), a subset of artificial intelligence, to process and analyze large amounts of natural language data [8,9]. Moreover, deep learning NLP models can be further fine-tuned on a large variety of tasks that could reach performance on par or if not better than humans [10,11]. One of the most popular data sources used for NLP is social media, such as Twitter [12], Facebook [13], Sina Weibo, and Yahoo!, and online forums like Reddit [14], to name a few.

There is a growing volume of literature adopting NLP techniques to extract and analyze social media data for PHS including monitoring public sentiments and health behaviors, predicting a pandemic, and detecting misinformation [1,14-18]. However, there could be potential bias from using social media data due to selected data sets that could overlook underrepresented population groups (generalizability) or contain misinformation (validity) [19-21]. On the other hand, Open Source Intelligence, which includes published and broadcasted news reports, may play a central role in national security, including regarding health emergencies, which often are highly covered. However, such news sources have been less leveraged in the existing models and literature [19,22]. Varol et al [22] published one of the few pieces of literature analyzing news coverage of CNN and the Guardian by using clinical and biomedical NLP models from the Spark NLP for Healthcare library to understand adverse reactions to drugs and vaccines that are used to combat the virus [22].

Although most PHS studies applying NLP on open-source data from publicly available sources focused on the population in the community [1,6,23], less is understood about the frontline health workers who are essential for the provision of health care services yet most directly affected by the pandemic. Compared with the general population, health workers were more susceptible to infections due to frequent contact with infected patients [24]. In addition to higher rates of infection and death, health workers also faced challenges from discontinued education or training, financial hardship, and impaired health and wellness due to the pandemic, which could further negatively affect the quality of services and patient outcomes [25]. Hence, it is necessary to have a timely understanding of the different impacts of COVID-19 on health workers in order to construct a targeted intervention.

In this study, we leveraged millions of worldwide news articles in English from publicly available sources collected by the World Health Organization (WHO) Epidemic Intelligence from Open Sources (EIOS) database. We developed an NLP framework named DeepCovid that automatically finds then summarizes relevant articles from EIOS. DeepCovid was designed by a joint team of computer scientists, medical doctors, and population health experts. Beyond the COVID-19 use case, we present a framework that can be leveraged in other PHS applications and support health policymakers with strategic intelligence.

## Methods

**Figure 1:**
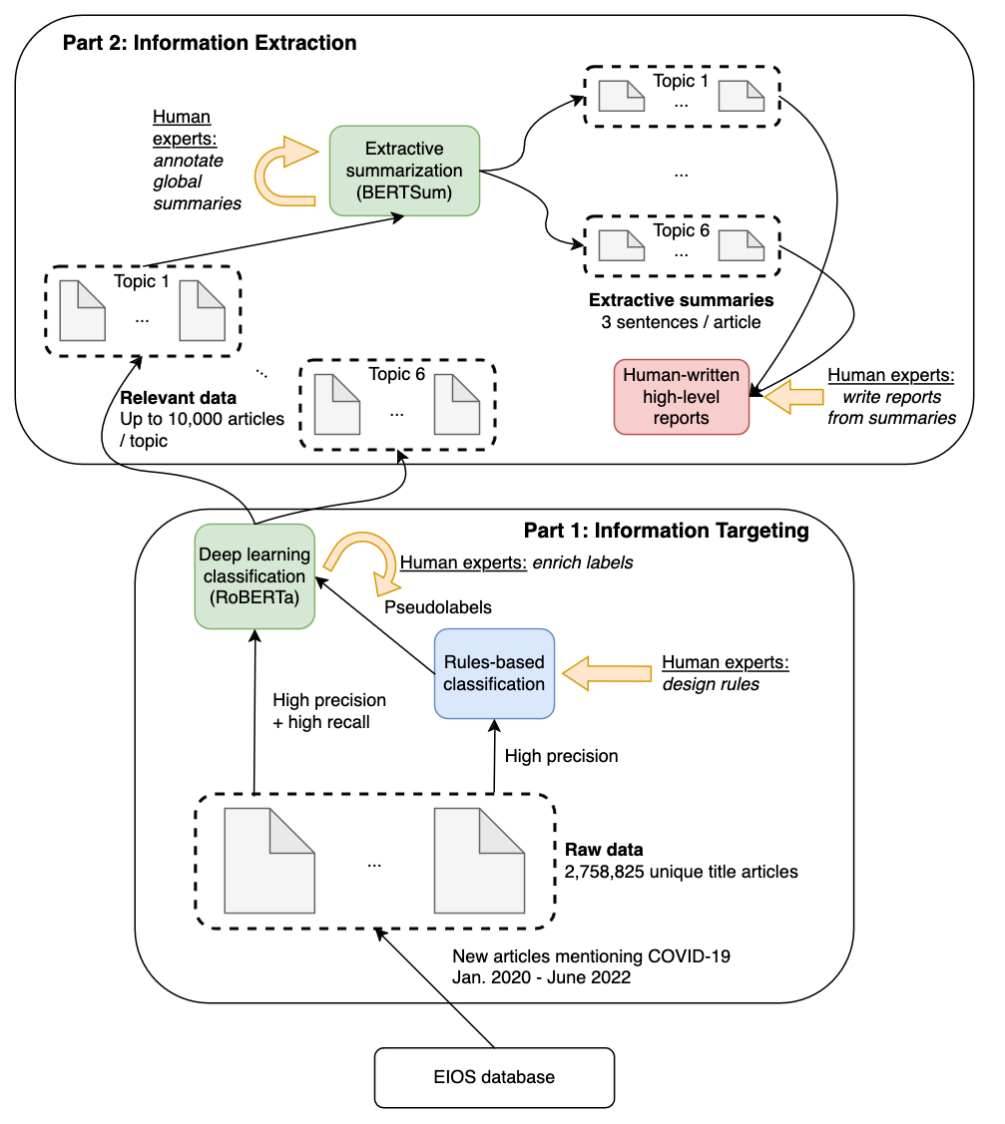
DeepCovid model architecture overview (read from bottom to top), with colored blocks corresponding to machine learning models and gold arrows indicating actions necessitated from human experts. BERT: Bidirectional Encoder Representations from Transformers; EIOS: Epidemic Intelligence from Open Sources; RoBERTa: Robustly Optimized BERT Pretraining Approach.

In this section, we describe each component of DeepCovid. The overall system, which aims to classify, arrange, and reduce a big volume of data comprising news articles, is pictured in Figure 1. The first part of the system aims to find relevant articles, and it trains and applies a deep learning classifier onto the database (information targeting). The resulting news articles move on to the next stage of information extraction, which aims to summarize relevant articles. An extractive summarization model summarizes each article into 3 sentences. Corresponding summaries are then analyzed by human experts to produce reports.

### Rules-Based Classification

After preliminary data cleaning, which included deduplication, we built inclusion rules for each of the 6 predetermined topics separately, validating choices through human assessment of precision. The end goal was to narrow the database to a set of relevant articles for each topic of interest: An article was kept if and only if it passed all rules for this topic. Our rules were independent from the lexical tagging already performed within EIOS comprising health care professions and a COVID-19 category.

Rules were designed on both the article title and body. Rules rely on sets of manually identified keywords listed by domain experts and the logical operators OR and AND. Rules can be inclusive, meaning the article is kept if some of the keywords are present, or exclusive, discarding the article if it contains some keywords. There could be multiple such operators nested to form a single rule, such as *one keyword among keywords_list_1 in the body OR one keyword among keywords_list_2 in the body AND two keywords among keywords_list_3 in TITLE.* When working on the article text body, some rules scan for at least one sentence being positive, in which case the entire article is considered to have passed the given rule. We list the set of rules for each topic in Multimedia Appendix 1.

After filtering news articles through rules, we also mapped each article to a unique country among its sets of countries tagged by EIOS. On average, each article has 2.07 such initial country tags from EIOS. Reducing to a single country tag reduces noise and enables the creation of pools of relevant articles per country, which allows further synthesis of key information. When country names are present in the title or first article sentence, we mapped the article to the most frequent such country. Otherwise, we used a deep learning embedding approach. Specifically, we collected all LOC (denoting location) and PERSON (denoting a person’s name [eg, Barack Obama]) entities from the *spacy* library [26] in the article body, concatenated them, and encoded them with a Robustly Optimized Bidirectional Encoder Representations from Transformers (BERT) Pretraining Approach (RoBERTa) model [27]. RoBERTa CLS token embeddings then yield a representation with the desired behavior. We also encoded each country name with the same RoBERTa model and returned the country whose representation maximizes the cosine similarity with the article representation.

### Deep Learning–Based Classification

#### Architecture

The aforementioned rules-based classification provides a hard assignment for each article to a predetermined topic: Either the article is marked as relevant for at least one topic, or it is not, and it is discarded. There are major limitations to such an algorithm: Articles found as positive may be irrelevant as the presence of key terms does not entice a core focus of the topic of interest (false positives), and many relevant articles might have been missed (false negatives), for instance if no keyword is found within the article. Designing a system that avoids false positives was, however, out of the scope for this study. To tackle false negatives and improve recall, we built a classifier based on a deep learning model: Such models learn a dense vector representation of a news article that can be used for further classification of the article without being limited by the specific choice of words in the text. The classifier assigns to each article a soft probability that it is positive for each of the topics of interest in a multilabel binary classification fashion.

**Figure 2:**
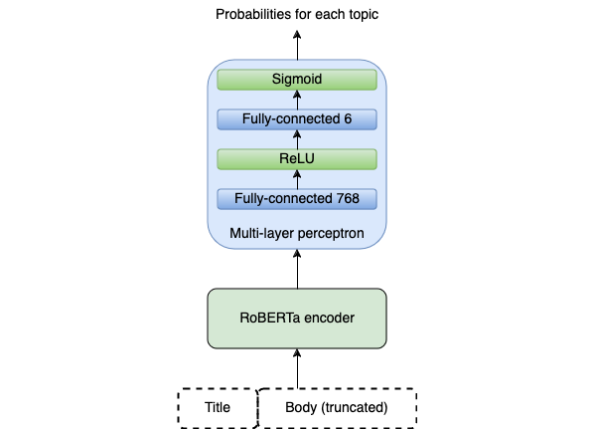
Deep learning classifier architecture. ReLU: rectified linear unit; RoBERTa: Robustly Optimized BERT Pretraining Approach.

Following the success of large pretraining language models for natural language understanding [27-32], we selected RoBERTa-base [27] as a backbone language model. As input to RoBERTa, we concatenated the title and article body and truncated the resulting string to the maximum input length of 512 tokens. We added 2 fully connected layers, one with a hidden size of 768, followed by rectified linear unit (ReLU) nonlinearity [33], and the last one with a hidden size of 6 (number of topics of interest), followed by Sigmoid as the final output layer. An overview of the classifier architecture is shown in Figure 2.

#### Deep Classification Labels Construction

We trained the deep classifier with the multilabel binary cross-entropy loss using the rules-based classification hard assignments as labels. Due to the very large volume of articles and a low expected fraction of relevant articles, it is unrealistic to collect human annotations for a training set for classification. We made a 90%-10% random training-validation split over the 28-month period from January 2020 to April 2022. The imbalanced nature of the classification problem was challenging: There were a few thousand positives but a few million negatives. Initial labels were provided by rules-based hard assignment. To ensure clean positive labels, volunteer human experts scanned all positive articles and discarded irrelevant ones. From the resulting labeling, in the training set, we kept all positive samples but randomly subsampled 100,000 negative articles. No class rebalancing was performed on the validation set.

After training the first version of the model, we made an inference on the entire 28-month data set and sorted down articles by decreasing the predicted probability for each topic. Human experts were asked to review articles initially flagged as negative but among the top 500 highest predicted scores, which significantly augmented the number of positive labels. After this labeling enrichment, we ended with 6512 positive articles in the training set, leaving 100,000 negative articles (positive ratio: 6.11%). The final validation set consisted of 270,324 articles, including 723 positives and 269,601 negatives (positive ratio: 0.27%). We fine-tuned the deep classifier again with these augmented sets of labels.

In both fine-tuning rounds, we trained for 5 epochs, with a learning rate of 1e^–5^ and the Adam optimizer [34]. We used a batch size of 4 and evaluated the model every 5000 optimization steps. We warmed up the learning rate linearly over the first 5% training steps, then linearly decreased it to 0 in the following 95% steps. We measured performance with the area under the receiver operating characteristic (ROC) of the area under the curve (AUC) metric and performed early stopping, saving a new checkpoint whenever the validation AUC improved.

For inference and real-time use of the system, we kept all articles with a predicted probability either high enough (>0.95) or within 3 times the number of articles flagged as positives by the rules-based model for each topic.

### Extractive Summarization

Once the number of relevant articles has been narrowed through article-level classification, the goal of summarization is to give the user a high-level, concise summary of the key information present in the article. Despite recent progress in abstractive summarization, such models are known to be prone to hallucinations [35-37], a problem partly fueled by the fact that commonly used fine-tuning data sets themselves contain hallucinations [35,38]. Given the critical use case for DeepCovid, we decided to use an extractive summarization model [39,40]. In the following sections, we describe how we built 2 sets of extractive summarization labels to fine-tune DeepCovid.

#### Summarization Labels

Unlike the classification model, the summarization model operates on a manageable volume of news articles. Therefore, we decided to collect human annotations. We asked volunteer graduate students, all fluent English speakers, to label articles among the positives from the rules-based classification. Annotators were asked to highlight between 1 and 3 sentences forming a *global extractive summary* of the article. We obtained annotations for 4062 unique articles, with at least 300 annotations per topic.

To ensure human agreement, we collected labels from 3 different humans for each article for 1 of the topics. Human labels were lists of selected sentences, and we used Fleiss kappa [41] and Gwet AC1 [42] as metrics to measure agreement. The two are complementary, as Gwet AC1 does not account for chance, unlike Fleiss kappa. The Fleiss kappa was 34.23, and for this metric, random agreement stands at 0. The Gwet AC1 was 83.80, with a random agreement of 19.16 in our setup. These values were in line with reported results in extractive summarization research [43], and we concluded that the labelers agreed enough in this task for us to collect a single human annotation per data point. The distribution of sentence positions selected by the human annotators is shown in Multimedia Appendix 2.

On top of these human global summarization labels, we also made use of pseudolabels from the rules-based classification model to obtain topic-focused summarization labels. Indeed, all but Topic 4 rules make use of sentence-level inclusion rules (eg, the article is kept if at least 1 sentence contains 1 of the keywords). We treated such sentences as pseudolabels for extractive summarization and built a set of 7491 pseudolabels.

#### Summarization Fine-Tuning

We used BERTExt, a state-of-the-art extractive summarization model, as a sentence selection model [44]. Since our data had uppercase and lowercase letters, we used bert-base-cased as the backbone pre-trained BERT model in BERTExt, downloading it from the HuggingFace *transformers* library [45]. To fine-tune jointly for both sets of the aforementioned labels, we doubled the prediction head. This means that the model assigned 2 probabilities to each sentence of the article: 1 to predict if the sentence should be in the global summary and 1 to predict if the sentence should be in the topic-focused pseudosummary. Each prediction head gave us a ranking of sentences, sorted by decreasing predicted probabilities. We also summed both predicted probabilities and sorted sentences by decreasing sum. We output the first 3 sentences of this final ranking as the final predicted summaries. These summaries capture both a flavor of the global sense of the article and a flavor of the topic-specific information contained in the article.

Given the small volume of available labels from each label source, we fine-tuned BERTEx on the CNN-DailyMail (CNN/DM) data set first [46]. CNN/DM is arguably the most widely used data set in both extractive and abstractive summarization [46-48] and comprises more than 300,000 news articles with corresponding human-written highlights (bullet points) serving as abstractive summaries. Following prior work [49], we built extractive summary labels by greedily matching each bullet point summary sentence to the source sentence maximizing Recall-Oriented Understudy for Gisting Evaluation (ROUGE)-1 with it.

We randomly sampled 1800 (300 for each topic) articles to form a validation set, leaving the remaining 6708 articles marked positive by rules-based classification as a training set. We only included articles with human global summarization annotations in the validation set. Since articles in the training set may have lacked either the topic-specific or global summary, we only computed training loss on the available labels, masking out predictions in the case of missing labels. We trained the model for 10 epochs and evaluated the model every epoch. We used the mean of ROUGE-1, ROUGE-2, and ROUGE-L as metrics [50] and performed early stopping. We trained with the Adam optimizer with a learning rate of 1e^–5^ [34]. We warmed up the learning rate linearly over the first 10% training steps, then linearly decreased it to 0 in the following 90% steps. The same optimization procedure was used when performing the initial fine-tuning on the CNN/DM data set.

### System Flow

**Figure 3:**
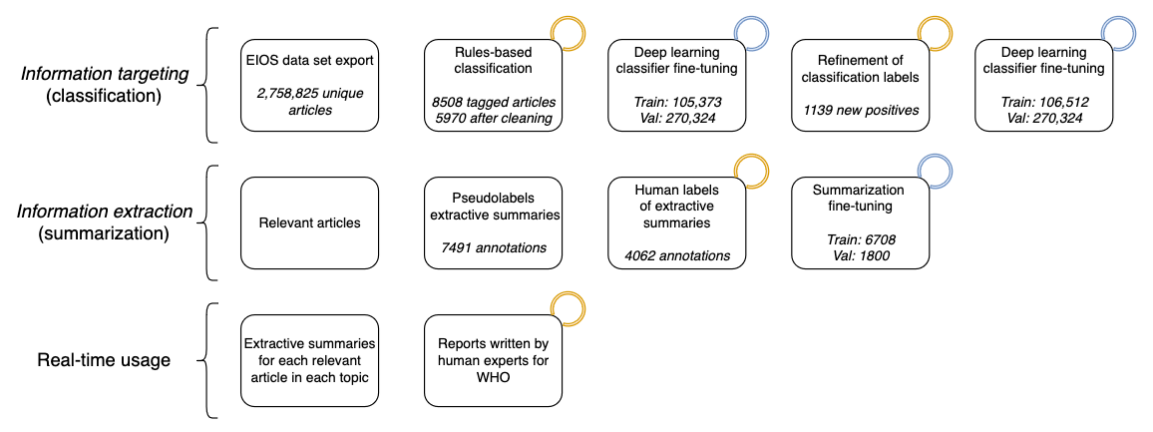
Flowchart for DeepCovid showing the step-by-step process transforming a raw data set of 2.8 million news articles (top left) to high-level reports (bottom-right). Boxes with an orange top-right ring indicate the need for human annotation, while boxes with a blue ring correspond to training a deep learning model. EIOS: Epidemic Intelligence from Open Sources; Val: validation; WHO: World Health Organization.

In Figure 3, we show the interplay of each component of our DeepCovid system, with the corresponding data subset size. Our system automatically narrows down the raw data of 2.8 million articles to topic-focused short summaries of highly relevant articles. 

## Results

### Data Set

We used data from the EIOS database ranging from January 1, 2020, to June 30, 2022 (totaling 30 months). EIOS tracks news articles on the web from more than 12,000 publicly available news outlets in more than 200 countries and territories. Data were filtered for the English language and with keywords relevant to the health workforce (Multimedia Appendix 3). Each article in the resulting data set was tagged by EIOS in-house lexical classification patterns with at least 1 matching keyword (there could be more). Verification using the *langid* package confirmed that more than 99.8% of the articles were indeed in English [51]. The initial data set contained 3,235,657 news articles from 3472 different unique sources and tagged with 243 different locations. After removing duplicate articles based on the title, our final working data set contained 2,758,825 unique news articles. Further statistics on the working data set can be found in Multimedia Appendix 4.

### Information Targeting Through Article-Level Classification

The information targeting component of DeepCovid serves the purpose of reducing noise in the data set to narrow it down to only the relevant articles for each of the 6 topics of interest from WHO. Namely, these topics of interest are (1) policy regarding management of and investments in the health workforce, (2) education of health workers, (3) vaccination of health workers, (4) strikes and industrial actions by health workers, (5) mental health issues of health workers, and (6) health worker infections and deaths.

We first created a rules-based classification, and the outputs were used to train the deep learning–based classification component of DeepCovid. Rules are lexical matches, with inclusion and exclusion criteria, and are defined at both the title level and article body level. The detailed list of rules for each topic can be found in Multimedia Appendix 1. This rules-based classification component was built to improve the precision of EIOS-retrieved articles and reduce the volume of irrelevant articles. We assessed the performance of the rules-based classification using human evaluation. Among articles marked as positive by the rules-based system, we subsampled 50 articles randomly for each topic and asked a human domain expert to label them as relevant with regards to the topic or not. Three human experts volunteered, and each human rater was assigned 2 different topics.

Rules-based classification number of positives (N) per topic, relevancy rate (precision), and overlap between topics are shown in Table 1. Overall, the rules-based classification identified a very small fraction of articles (8508 in total, 0.053% on average across the 6 topics) with a high fraction of them (258/300, 86%) being marked relevant by humans, proving its high precision. However, we highlight that this high precision is achieved after 2 rounds of article selection through lexical rules (the rules in EIOS and the subsequent proposed rules by us), and it is therefore not the “true” precision that would be achieved on a large random sample of articles crawled from the web. We also acknowledge the inherent subjectivity in human assessment of relevancy, and judgments may vary from one human to another [52,53]. Besides, as seen in the confusion matrix, the overlap between topics is small: for instance, of 1125 articles identified for Topic 1, 9 (0.8%) of them also belong to Topic 2.

**Table 1:** Rules-based classifier for the 28 million-article data set from January 2020 to April 2022.

Topic	Positive rate, n (%)^a^	Relevant, n (%)^b^	Overlap between topics^c^, n					
			Topic 1	Topic 2	Topic 3	Topic 4	Topic 5	Topic 6
Topic 1	1125 (0.041)	45 (90)	—^d^	9	0	5	9	1
Topic 2	1706 (0.062)	45 (90)	9	—	3	6	21	5
Topic 3	2077 (0.075)	44 (88)	0	3	—	14	22	81
Topic 4	1102 (0.040)	44 (88)	5	6	14	—	56	4
Topic 5	1444 (0.052)	36 (72)	9	21	22	56	—	41
Topic 6	1331 (0.048)	44 (88)	1	5	81	4	41	—

^a^Marked relevant by the rules-based system; overall mean: 1464/28,000,000, 0.053%.

^b^The articles tagged by rules (among a random sample of 50) that were confirmed as relevant to the topic by human experts; overall mean: 43/50, 86%.

^c^Subset of articles that also belong to the topic listed in the column.

^d^Not applicable.

By construction, the rules-based classification identified a high *precision* subset of news articles (86% relevancy rate). However, it has no mechanism to ensure high *recall*, which is one of the motivations behind subsequently training the deep classifier. After training the first version of the deep classifier (tagged as the “initial model”), we made an inference on the entire data set and asked human evaluators to examine the articles that did not past the rules but among the top 500 highest predictions (the “Relevant” column). This corresponds to articles initially missed by the rules yet flagged as extremely relevant by the deep learning model. Such a relabeling process enabled us to enrich the rules-based labels with human annotations while avoiding a human inspection of 2.8 million news articles. Human annotation for this phase was done with the same volunteers as in the previous phase. We then trained the deep classifier again (tagged as the “final model”) with the cleaner labels and evaluated it with the AUC. To understand what relative ranking the deep classifier assigned to articles marked positive by rules, we also report the Precision@k.N and Recall@k.N, where N is the number of articles marked positive by the rules-based process and k is an integer (eg, 1, 2, or 10). Table 2 reports the relevance and performance of the results. 

The deep learning classifier achieved a consistent and very high AUC across topics (88.54 on average), attesting to both the strength of the signal singled out by the rules and the capacity of the deep classifier to accurately learn it. Indeed, if human-curated lexical rules were poorly designed, a high-capacity pretrained language model would struggle to capture their topic and linguistic style such as words, word patterns, and phrases. The high percentage of relevant negatives among high prediction scores also shows promising capacity in the model to ensure higher recall.

**Table 2:** Deep learning classifier performance on the classification validation set.

Topic	Relevance of the initial model			Performance of the final model					
	High-score negatives^a^, n	Relevant, n (%)^b^	ROC-AUC^c^		PR-AUC^d^	Prec@^e^ 2N	Prec@ 10N	Rec@^f^ 2N	Rec@ 10N
Topic 1	309	255 (82.5)	99.33		22.77	20.00	5.87	40.00	58.67
Topic 2	299	234 (78.3)	94.87		24.55	19.64	5.79	39.29	57.86
Topic 3	272	270 (99.3)	99.92		42.75	36.56	9.78	73.12	97.85
Topic 4	151	150 (99.3)	99.98		84.62	48.75	10.00	97.50	100.00
Topic 5	220	161 (73.2)	99.86		39.65	29.85	8.06	59.70	80.60
Topic 6	230	227 (98.7)	99.93		68.72	42.13	9.81	84.26	98.15
All topics, mean	216.17	168.93 (88.5)	98.98		47.21	32.82	8.22	65.65	82.19

^a^Articles initially missed by the lexical rules (negatives) but were among the top 500 highest predicted score by the deep learning model.

^b^High-score negatives identified as relevant to the topic by human experts.

^c^ROC-AUC: area under the receiver operating characteristic curve.

^d^PR-AUC: area under the precision recall curve.

^e^Prec@: precision scores at different thresholds.

^f^Rec@: recall scores at different thresholds.

### Information Extraction With Extractive Summarization

The subsequent module of DeepCovid tackled information extraction, which identified the key takeaways among articles previously marked as relevant. We evaluated the summarization performance with the standard ROUGE metric [50], averaging its 3 commonly used versions ROUGE-1/2/L. We report the mean ROUGE achieved by the extractive summarization component of DeepCovid on each topic in Table 3, alongside ablated versions with which the model had access to less training supervision. We experimented with sentences selected by lexical rules (“selected sentences”), sentences annotated by humans (“human sentences”), and fine-tuning on the CNN/DM news summarization data set.

CNN/DM means that the model was fine-tuned on the news summarization benchmark CNN/DM first [46]. *Selected* refers to the model being fine-tuned with sentences flagged by the rules-based classification as labels (conveying a pseudosummary focused to each topic), and *human* refers to fine-tuning with human annotations that were designed to build a global summary. In practice, we used the model fine-tuned with all 3 options (denoted as the *final model*), even though it reached slightly less performance than *CNN/DM + human*, as we found its predicted summaries were more focused toward the topics of interest.

**Table 3:** Extractive summarization Recall-Oriented Understudy for Gisting Evaluation (ROUGE) results (mean of ROUGE-1, ROUGE-2, and ROUGE-L).

Model supervision^a^	Topic 1	Topic 2	Topic 3	Topic 4	Topic 5	Topic 6	Mean
None (random model weights)	21.90	19.76	27.65	25.37	25.35	25.34	24.23
Selected sentences	45.41	37.27	49.96	50.77	47.20	37.79	44.73
Human sentences	47.61	38.22	48.67	49.58	48.71	37.96	45.13
Selected + human sentences	52.15	38.49	47.19	47.78	51.85	42.00	46.58
CNN/DM^b^	45.33	36.80	45.86	48.57	48.19	35.81	43.43
CNN/DM + selected sentences	44.71	28.69	44.65	40.08	44.82	35.55	39.75
CNN/DM + human sentences	53.97	43.91	49.75	49.08	55.61	43.08	49.23
CNN/DM + selected sentences + human sentences (final model)	53.76	40.74	49.08	47.55	52.45	42.99	47.76

^a^Signal with which the extractive summarization model was trained.

^b^CNN-DM: CNN-DailyMail.

## Discussion

### Main Findings

The challenge posed by the pandemic offered an opportunity to improve PHS through the use of innovative NLP techniques [7]. Our newly developed framework DeepCovid has demonstrated how to semi-automatically extract precise, targeted news information on health workers concerning the COVID-19 pandemic. Leveraging a global, million-article scale news article database, this framework is able to provide global and population-level information on how COVID-19 impacts health workers that traditional methods (eg, survey, media monitoring) may not be able to do in a short time. With a generic and reusable method that can deal with a high volume of news articles published worldwide, DeepCovid can be used for any health care–related events such as a future similar pandemic and potentially be extended for events beyond the scope of health care, such as financial crises. The DeepCovid framework can assist policymakers with providing fast responses to future similar public health concerns.

Putting DeepCovid in place only requires 4 human actions (see Figure 1): (1) design classification rules to narrow the data set to relevant articles, (2) relabel (some of) the resulting positive and negative articles, (3) label a small set of global extractive summaries to seed the summarization model, and (4) finally, aggregate extractive summaries into reports. All 4 steps require a moderate volume of work from human workers, on the order of a few hours to a few days from 2 humans, which is several orders of magnitude lower than what would be required to manually go through such a scale of data as that which we applied the system, proving the efficiency of DeepCovid. Furthermore, a simpler version of DeepCovid bypassing human actions (2) and (3) leads to a system with reasonable performance, as the key human interventions are the initial (1) and final (4) ones.

Existing work using machine learning to address system-level challenges arising from the COVID-19 pandemic does not cover the multiple impacts of the pandemic on the worldwide health workforce. We note one study that predicted the mental health of Chinese medical workers with logistic regression analysis [54]. In the realm of NLP applications, another study predicted sentiment from tweets by Indian citizens using BERT to assess public opinion during a lockdown [55]. Another paper leveraged long short-term memory networks to predict the number of deaths from WHO data in 3 countries [56]. The most relevant system to ours is CO-Search [57], which is a multicomponent deep learning pipeline enabling the user to find relevant documents with regards to a query; answer questions; and summarize them, leveraging scientific publications from the CORD-19 challenge [58]. However, the CO-Search input data are wildly different from the news data in our study.

With a data set the scale of EIOS, topic-specific precision and recall evaluation remain an open research issue. We showed that DeepCovid rules-based classification may reach high precision through human evaluation, but this is at the cost of 2 rounds of lexical filtering (EIOS and DeepCovid), and human precision evaluation itself is not perfect due to the subjectivity among raters. DeepCovid proposes a mechanism to boost recall of relevant articles through deep learning, yet “true” recall remains impossible to measure as it would involve an extremely costly human inspection of 2.8 million articles. Language models like the ones used in DeepCovid are not equipped with semantical understanding of what classification rules are designed to capture and merely rely on statistical co-occurrence patterns, which enables relevant articles to be expanded with other articles containing similar topics albeit phrased with different lexicality. Striving for perfect precision and recall may need other, complementary tools to deep pretrained language models, such as knowledge graphs.

With regards to optimization, DeepCovid's double objective makes training in a single phase complicated. Document classification and extractive summarization are different types of tasks, and reducing them to a single model addressing both might compromise performance, motivating our choice to keep separate modules, each proven to be a leading approach, even though this adds some complexity and requires 2 separate training processes. Another limitation of our work lies in the fact that human intervention remains compulsory at steps (1) and (4).

Recent progress in large language models (LLMs), sometimes referred to as foundation models, such as GPT-3 [59] or GPT-4 [60], opens a new perspective. Since these models can perform many complicated tasks in few-shot in-context learning [61], or even zero-shot, including summarization [62], we believe that they hold great promise for automating the final step (4) and could synthesize and combine insights from the set of extractive summaries, even more so by decomposing report writing into a template of specific instructions, which has been shown to dramatically boost performance of these models [63]. Acting as agents, LLMs can work hand-in-hand with human experts to create new annotations in cases where annotations are scarce [64], which in turn can be successfully used to fine-tune smaller language models. However, we highlight that LLMs are not a silver bullet since they are hidden behind a paywall and may hallucinate subtly, generating false content that only seasoned domain experts would spot at first glance [65]. We leave the evaluation of the performance of LLMs to better streamline DeepCovid to future work. Emergent capabilities of LLMs such as reasoning [63,66,67] may also be explored for information targeting: from a classification perspective in order to build classifiers (potentially bypassing the construction of lexical rules) and for evaluation of classification precision. 

### Limitations

The findings of this study should be interpreted in light of several limitations. Although DeepCovid can be a useful tool to extract information from open-source data and assist policymakers during the process of policymaking, it should not be the sole tool for decision-making. What is more important and essential to fight future similar emerging diseases is cross-jurisdictional and cross-functional coordination and collaboration [21].

First, our study was restricted to English-only news articles. This decision was based on the abundance of English sources compared with other languages. From the perspective of the data source, a model that is trained on English-only news articles is likely to miss information from non-English reported news, resulting in biased samples and underestimating the pandemic impact on underrepresented groups. Technically, a multilingual version of DeepCovid is very feasible. It would involve replacing each deep learning component with a multilingual model version (eg, mBERT instead of BERT for the information targeting encoder), which we leave to future work. With model improvement that is compatible with more languages and modalities, DeepCovid will better provide representative information for the global population.

Another limitation lies in the need for expert annotations to bootstrap fine-tuning for each component. This is time-consuming but critical for the final system performance. We envision that new capabilities of LLMs would in the future enable us to replace human annotators with LLM-generated annotations instead, particularly with powerful LLMs such as GPT-4. However, although annotation time would be reduced, using the GPT-4 application programming interface still bears a significant cost. Besides, annotations generated by LLMs would still need to be validated by human experts.

Although broad and valuable, the data set contains a relatively narrow type of news coverage, hence additional insights could be gained by expanding the data sources to social media channels and broadening the format to multimedia content such as videos. The data and findings are impacted by specific strategies for open-source collection that can manifest with, for example, underrepresentation of some countries. Additionally, the current work has not included the identification and exclusion of fake news or reporting biases. Further improvement focusing on bias removal techniques will be needed in order to remove bias from the training data inherited by DeepCovid.

Last, we highlight that DeepCovid synthesizes post hoc information, as news articles usually cover recent (yet, past) events. Findings from DeepCovid may be most useful if acted on early and may be of little use to predict future events.

### Conclusion

In this study, we introduced the DeepCovid system. Relying on 2 deep learning–powered components, DeepCovid automatically finds topic-focused relevant news articles among millions of candidates before writing succinct extractive summaries from them. We validated the performance of each component through both human evaluation and automatic metrics, confirming the high performance of the system: Information targeting can reach an AUC in the 98-99 range, and information extraction has an average ROUGE score of 47-48. Core elements of DeepCovid were successfully used to power the Workforce Intelligence from Open Sources project commissioned by the WHO. The findings are to be published in a separate paper. DeepCovid methodology also makes it suitable for use cases other than COVID-19, for instance global events with large news coverage from open sources.

## Appendix

Multimedia Appendix 1 Rules-based classification.

Multimedia Appendix 2 Extractive summarization labels.

Multimedia Appendix 3 EIOS classification.

Multimedia Appendix 4 Data set statistics.

## Abbreviations

AUC: area under the receiver operating characteristic curve
BERT: Bidirectional Encoder Representations from Transformers
CNN-DM: CNN-DailyMail
EIOS: Epidemic Intelligence from Open Sources
LLM: large language model
NLP: natural language processing
PHS: public health surveillance
ReLU: rectified linear unit
RoBERTa: Robustly Optimized BERT Pretraining Approach
ROC: receiver operating characteristic
ROUGE: Recall-Oriented Understudy for Gisting Evaluation
WHO: World Health Organization
