# Supplementary material for: Targeting COVID-19 and Human Resources for Health News Information Extraction: Algorithm Development and Validation
Source: JMIR AI. 2024 Oct 30;3:e55059. doi: 10.2196/55059 (PMC11561429; doi:10.2196/55059)
Supplement: Multimedia Appendix 1 [file ai_v3i1e55059_app1.docx]

# Rules-based classification

Here, we list all lexical rules derived for the rules-based classification of DeepCovid. All rules are created by domain experts co-authors of this paper.

Rules applied for all topics:

1. If several articles share the same title, keep the first article in terms of publication date.
2. Remove articles with at least one sentence longer than 100 words

**Topic 1: Policy, management, regulation and investment decisions for surge capacity, including on hiring, deploying and redistributing health workers to address pandemic-induced shortages**

1. (**Inclusion**) Keep articles with the **title** containing at least one **topic-specific** keyword from:

   {*bill, law, legislat, act , investment plan, investment program, budget, regulatio, government, minister, ministry}*
2. (**Inclusion**) Keep articles with at least a sentence in the **body** containing at least one **topic-specific** keyword from:

   {*bill, law, legislat, act, investment plan, investment program, budget, regulatio}*

   **AND** at least one **topic-specific** keyword from:

   {*government, minister, ministry*}
3. (**Inclusion**) Keep articles with at least a sentence in the **body** containing at least one **professions** keyword from:

   {*health specialist, health provider, health professional, health practitioner, health doctor, health worker, health personnel, health staff, healthcare specialist, healthcare provider, healthcare professional, healthcare practitioner, healthcare doctor, healthcare worker, healthcare personnel, healthcare staff, health care specialist, health care provider, health care professional, health care practitioner, health care doctor, health care worker, health care personnel, health care staff, medical specialist, medical provider, medical professional, medical practitioner, medical doctor, medical worker, medical personnel, medical staff, physician, general practitioner, therapist, nurse, midwife*, caregiver, care provider, gastroenterologist, surgeon, dentist, psychiatrist, pharmacist, ophthalmologist, cardiologist, anaesthesiologist, anesthesiologist, dermatologist, endocrinologist, geriatrician, haematologist, hematologist, nephrologist, neurologist, oncologist, otolaryngologist, pulmonologist, paediatrician, pediatrician, radiologist, urologist*}

   **AND** at least one **topic-specific** keyword from:

   {*salary, allowance, overtime, pay, risk insurance, incentiv, shortage, redeploy, surge capacity, hire, hiring, repurpose, redeploy, redistrib, recruit, remuneration, spend, spent, retire}*

**Topic 2: Disruptions, adaptations, and acceleration of undergraduate and post graduate health education.**

1. (**Exclusion**) Remove articles with “NEWS FEED” in the body.
2. (**Inclusion)** Keep articles with at least one sentence in the **body** containing at least one **topic-specific** keyword from:

   {*medical student, medical graduate, medical studies, medical school, medical program, medical college, medical trainee, medical undergraduate, medical degree, foreign medical graduate, foreign medical student, international medical graduate, medical student, nursing student, nursing studies, nursing school, nursing program, nursing college, nursing trainee, nursing degree, dental student, dental studies, dental program, pharmaceutical student, pharmaceutical studies, pharmaceutical program*}

   **AND** at least one **topic-specific** keyword from:

   {*graduate early, graduating early, early graduation, early exit, early registration, fast-track, fast track, final year, e-learning, online learning, recruit, suspend, cancel, exam, exam., exam,, online, seats, volunte, visas, visa, not permitted, permit, license, admission, capacity, accelerat, placement*}

**Topic 3: Barriers, enablers, and coverage of COVID-19 vaccination for health and care workers**

1. (**Inclusion**) Keep articles with the **title** containing at least one **topic-specific** keyword from:

   {*vaccin, booster, jab, shot, Pfizer, BioNTech, Moderna, Sinovac, Janssen, Johnson & Johnson, AstraZeneca, Covishield, Covaxin, Sinovac, Sinopharm, Sputnik*}

   **AND** at least one **professions** keyword from:

   {*health specialist, health provider, health professional, health practitioner, health doctor, health worker, health personnel, health staff, healthcare specialist, healthcare provider, healthcare professional, healthcare practitioner, healthcare doctor, healthcare worker, healthcare personnel, healthcare staff, health care specialist, health care provider, health care professional, health care practitioner, health care doctor, health care worker, health care personnel, health care staff, medical specialist, medical provider, medical professional, medical practitioner, medical doctor, medical worker, medical personnel, medical staff, physician, general practitioner, therapist, nurse, midwife*, caregiver, care provider, gastroenterologist, surgeon, dentist, psychiatrist, pharmacist, ophthalmologist, cardiologist, anaesthesiologist, anesthesiologist, dermatologist, endocrinologist, geriatrician, haematologist, hematologist, nephrologist, neurologist, oncologist, otolaryngologist, pulmonologist, paediatrician, pediatrician, radiologist, urologist*}
2. (**Inclusion**) Keep articles with at least one sentence in the **body** following one these two patterns:
   1. The sentence has at least one **topic-specific** keyword from:

      {*vaccin, immuniz, immunis, booster, jab, shot, Pfizer, BioNTech, Moderna, Sinovac, Janssen, Johnson & Johnson, AstraZeneca, Covishield, Covaxin, Sinovac, Sinopharm, Sputnik*}

      **AND** at least one **professions** keyword from:

      {*health specialist, health provider, health professional, health practitioner, health doctor, health worker, health personnel, health staff, healthcare specialist, healthcare provider, healthcare professional, healthcare practitioner, healthcare doctor, healthcare worker, healthcare personnel, healthcare staff, health care specialist, health care provider, health care professional, health care practitioner, health care doctor, health care worker, health care personnel, health care staff, medical specialist, medical provider, medical professional, medical practitioner, medical doctor, medical worker, medical personnel, medical staff, physician, general practitioner, therapist, nurse, midwife*, caregiver, care provider, gastroenterologist, surgeon, dentist, psychiatrist, pharmacist, ophthalmologist, cardiologist, anaesthesiologist, anesthesiologist, dermatologist, endocrinologist, geriatrician, haematologist, hematologist, nephrologist, neurologist, oncologist, otolaryngologist, pulmonologist, paediatrician, pediatrician, radiologist, urologist*}

      **AND** at least one **number** which does not follow any of the following patterns:

      *{-number, number-, number+space+min, number+space+sec, number/, number year old, number years old, number,+space, number:, letter+number, number+space+letter, number+space+hour, number+space+h, month abbreviated+space+number, number+space+month abbreviated, month abbreviated, number}*
   2. The sentence has at least one **topic-specific** keyword from:

      {*vaccin, immuniz, immunis, booster, jab, shot, Pfizer, BioNTech, Moderna, Sinovac, Janssen, Johnson & Johnson, AstraZeneca, Covishield, Covaxin, Sinovac, Sinopharm, Sputnik*}

      **AND** at least one **professions** keyword from:

      {*health specialist, health provider, health professional, health practitioner, health doctor, health worker, health personnel, health staff, healthcare specialist, healthcare provider, healthcare professional, healthcare practitioner, healthcare doctor, healthcare worker, healthcare personnel, healthcare staff, health care specialist, health care provider, health care professional, health care practitioner, health care doctor, health care worker, health care personnel, health care staff, medical specialist, medical provider, medical professional, medical practitioner, medical doctor, medical worker, medical personnel, medical staff, physician, general practitioner, therapist, nurse, midwife*, caregiver, care provider, gastroenterologist, surgeon, dentist, psychiatrist, pharmacist, ophthalmologist, cardiologist, anaesthesiologist, anesthesiologist, dermatologist, endocrinologist, geriatrician, haematologist, hematologist, nephrologist, neurologist, oncologist, otolaryngologist, pulmonologist, paediatrician, pediatrician, radiologist, urologist*}

      **AND** at least one **topic-specific** keyword from:

      {*refus, resistan, hesitan, inequit, distribution, inequality, rollout, maldistribution, side effect, allergic reaction, mandate, required*}
3. (**Exclusion**) Remove articles with at least one sentence in the **body** containing at least one **topic-specific** keyword from:

   {*vaccin, immuniz, immunis, booster, jab, shot, Pfizer, BioNTech, Moderna, Sinovac, Janssen, Johnson & Johnson, AstraZeneca, Covishield, Covaxin, Sinovac, Sinopharm, Sputnik*}

   **AND** at least one **professions** keyword from:

   {*health specialist, health provider, health professional, health practitioner, health doctor, health worker, health personnel, health staff, healthcare specialist, healthcare provider, healthcare professional, healthcare practitioner, healthcare doctor, healthcare worker, healthcare personnel, healthcare staff, health care specialist, health care provider, health care professional, health care practitioner, health care doctor, health care worker, health care personnel, health care staff, medical specialist, medical provider, medical professional, medical practitioner, medical doctor, medical worker, medical personnel, medical staff, physician, general practitioner, therapist, nurse, midwife*, caregiver, care provider, gastroenterologist, surgeon, dentist, psychiatrist, pharmacist, ophthalmologist, cardiologist, anaesthesiologist, anesthesiologist, dermatologist, endocrinologist, geriatrician, haematologist, hematologist, nephrologist, neurologist, oncologist, otolaryngologist, pulmonologist, paediatrician, pediatrician, radiologist, urologist*}

   **AND** at least one keyword from:

   {*patient, public, warning, control-study, training, preliminary data, administer*}

**Topic 4: Industrial action by health workers.**

1. (**Inclusion**) Keep articles with the **title** containing at least one **topic-specific** keyword from:

   {*strike, protest, industrial action*}

   **AND** at least one **professions** keyword from:

   {*health specialist, health provider, health professional, health practitioner, health doctor, health worker, health personnel, health staff, healthcare specialist, healthcare provider, healthcare professional, healthcare practitioner, healthcare doctor, healthcare worker, healthcare personnel, healthcare staff, health care specialist, health care provider, health care professional, health care practitioner, health care doctor, health care worker, health care personnel, health care staff, medical specialist, medical provider, medical professional, medical practitioner, medical doctor, medical worker, medical personnel, medical staff, physician, general practitioner, therapist, nurse, midwife*, caregiver, care provider, gastroenterologist, surgeon, dentist, psychiatrist, pharmacist, ophthalmologist, cardiologist, anaesthesiologist, anesthesiologist, dermatologist, endocrinologist, geriatrician, haematologist, hematologist, nephrologist, neurologist, oncologist, otolaryngologist, pulmonologist, paediatrician, pediatrician, radiologist, urologist*}
2. (**Exclusion)** Remove all articles with the **title** containing at least one **topic-specific** keyword from:

   {*virus strikes, student, teacher, lockdown, strike(s) vaccine, strike(s) COVID-19 vaccine, George Floyd, anti-vacc, black lives, trump rally*}

**Topic 5: Burnout, stress, and other mental health impacts on health workers**

1. (**Inclusion**) Keep all articles with the **title** containing at least one **professions** keyword from:

   {*health specialist, health provider, health professional, health practitioner, health doctor, health worker, health personnel, health staff, healthcare specialist, healthcare provider, healthcare professional, healthcare practitioner, healthcare doctor, healthcare worker, healthcare personnel, healthcare staff, health care specialist, health care provider, health care professional, health care practitioner, health care doctor, health care worker, health care personnel, health care staff, medical specialist, medical provider, medical professional, medical practitioner, medical doctor, medical worker, medical personnel, medical staff, physician, general practitioner, therapist, nurse, midwife*, caregiver, care provider, gastroenterologist, surgeon, dentist, psychiatrist, pharmacist, ophthalmologist, cardiologist, anaesthesiologist, anesthesiologist, dermatologist, endocrinologist, geriatrician, haematologist, hematologist, nephrologist, neurologist, oncologist, otolaryngologist, pulmonologist, paediatrician, pediatrician, radiologist, urologist*}
2. (**Inclusion**) Keep all articles with at least one sentence in the **body** containing at least one **topic-specific** keyword from:

   {*burnout, mental health, mental wellness, anxiety, depression, PTSD, traumatic stress, suicide, trauma, fatigued, overburdened, under stress, exhausted, stressed out, nervous breakdown, depressed, mental health issue, stress , stress,, burned out, overworked*} 

   **AND** at least one **professions** keyword from:

   {*health specialist, health provider, health professional, health practitioner, health doctor, health worker, health personnel, health staff, healthcare specialist, healthcare provider, healthcare professional, healthcare practitioner, healthcare doctor, healthcare worker, healthcare personnel, healthcare staff, health care specialist, health care provider, health care professional, health care practitioner, health care doctor, health care worker, health care personnel, health care staff, medical specialist, medical provider, medical professional, medical practitioner, medical doctor, medical worker, medical personnel, medical staff, physician, general practitioner, therapist, nurse, midwife*, caregiver, care provider, gastroenterologist, surgeon, dentist, psychiatrist, pharmacist, ophthalmologist, cardiologist, anaesthesiologist, anesthesiologist, dermatologist, endocrinologist, geriatrician, haematologist, hematologist, nephrologist, neurologist, oncologist, otolaryngologist, pulmonologist, paediatrician, pediatrician, radiologist, urologist*}
3. (**Exclusion**) Remove all articles with at least one sentence in the **body** containing at least one **topic-specific** keyword from:

   {*public anxiety, trauma nurse, trauma program, Surgery, Trauma, trauma center, trauma centre, trauma unit, trauma surgeon, trauma hospital, trauma service, trauma physician, in traumas, trauma patient, trauma care, trauma ICU nurse, traumatic times, trauma machine, Straumann, orthopedic trauma, trauma and orthopaedics, surgical trauma, emergency trauma, traumatic event, traumatic brain, trauma and emergency, facial trauma, mental health center, mental health centre, mental health team, mental health professional, governmental health, environmental health, mental health therapist, mental health provider, mental health nurse, mental health worker, mental health care provider, suicide mission, training in suicide, suicide nurse, people with, people deal, great depression, trauma protocol, Mental Health*}

**Topic 6: Health worker infection and deaths**

1. (**Inclusion**) Keep all articles with the **title** containing at least one **topic-specific** keyword from:

   {*dead, death, died, infect*, claimed lives, perished, passed away, lost live, decease, dying, illness*}

   **AND** at least one **professions** keyword from:

   {*health specialist, health provider, health professional, health practitioner, health doctor, health worker, health personnel, health staff, healthcare specialist, healthcare provider, healthcare professional, healthcare practitioner, healthcare doctor, healthcare worker, healthcare personnel, healthcare staff, health care specialist, health care provider, health care professional, health care practitioner, health care doctor, health care worker, health care personnel, health care staff, medical specialist, medical provider, medical professional, medical practitioner, medical doctor, medical worker, medical personnel, medical staff, physician, general practitioner, therapist, nurse, midwife*, caregiver, care provider, gastroenterologist, surgeon, dentist, psychiatrist, pharmacist, ophthalmologist, cardiologist, anaesthesiologist, anesthesiologist, dermatologist, endocrinologist, geriatrician, haematologist, hematologist, nephrologist, neurologist, oncologist, otolaryngologist, pulmonologist, paediatrician, pediatrician, radiologist, urologist*}
2. (**Inclusion**) Keep all articles with at least one sentence in the **body** containing at least one **topic-specific** keyword from:

   {*dead, death, died, infect*, contracted COVID-19, contracted the virus, tested positive, succumbed, claimed lives, perished, passed away, lost live, decease, dying, illness*}

   **AND** at least one **professions** keyword from:

   {*health specialist, health provider, health professional, health practitioner, health doctor, health worker, health personnel, health staff, healthcare specialist, healthcare provider, healthcare professional, healthcare practitioner, healthcare doctor, healthcare worker, healthcare personnel, healthcare staff, health care specialist, health care provider, health care professional, health care practitioner, health care doctor, health care worker, health care personnel, health care staff, medical specialist, medical provider, medical professional, medical practitioner, medical doctor, medical worker, medical personnel, medical staff, physician, general practitioner, therapist, nurse, midwife*, caregiver, care provider, gastroenterologist, surgeon, dentist, psychiatrist, pharmacist, ophthalmologist, cardiologist, anaesthesiologist, anesthesiologist, dermatologist, endocrinologist, geriatrician, haematologist, hematologist, nephrologist, neurologist, oncologist, otolaryngologist, pulmonologist, paediatrician, pediatrician, radiologist, urologist*}

   **AND** at least one **number** which does not follow any of the following patterns:

   *{-number, number-, number+space+min, number+space+sec, number/, number year old, number years old, number,+space, number:, letter+number, number+space+letter, number+space+hour, number+space+h, month abbreviated+space+number, number+space+month abbreviated, month abbreviated. number}*
3. (**Exclusion**) Remove all articles with at least one sentence in the **body** containing at least one **professions** keyword from:

   {*health specialist, health provider, health professional, health practitioner, health doctor, health worker, health personnel, health staff, healthcare specialist, healthcare provider, healthcare professional, healthcare practitioner, healthcare doctor, healthcare worker, healthcare personnel, healthcare staff, health care specialist, health care provider, health care professional, health care practitioner, health care doctor, health care worker, health care personnel, health care staff, medical specialist, medical provider, medical professional, medical practitioner, medical doctor, medical worker, medical personnel, medical staff, physician, general practitioner, therapist, nurse, midwife*, caregiver, care provider, gastroenterologist, surgeon, dentist, psychiatrist, pharmacist, ophthalmologist, cardiologist, anaesthesiologist, anesthesiologist, dermatologist, endocrinologist, geriatrician, haematologist, hematologist, nephrologist, neurologist, oncologist, otolaryngologist, pulmonologist, paediatrician, pediatrician, radiologist, urologist*}

   **AND** at least one **number** which does not follow any of the following patterns:

   *{-number, number-, number+space+min, number+space+sec, number/, number year old, number years old, number,+space, number:, letter+number, number+space+letter, number+space+hour, number+space+h, month abbreviated+space+number, number+space+month abbreviated, month abbreviated. number}*
