# Supplementary material for: Targeting COVID-19 and Human Resources for Health News Information Extraction: Algorithm Development and Validation
Source: JMIR AI. 2024 Oct 30;3:e55059. doi: 10.2196/55059 (PMC11561429; doi:10.2196/55059)
Supplement: Multimedia Appendix 2 [file ai_v3i1e55059_app2.docx]

# Extractive summarization labels

We give more details on the extractive summarization labels which we obtained from human volunteers. More specifically, with investigate whether a *lead bias* is present among sentences highlighted by humans, which corresponds to summarization labels in the news domain being concentrated among the first (or “lead”) sentences.


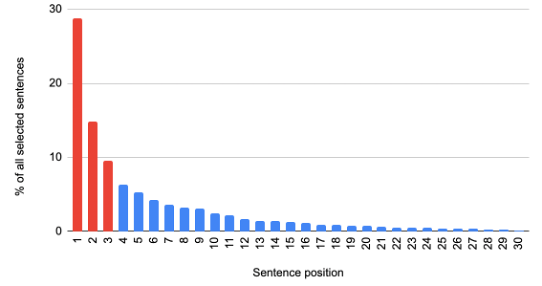


**Figure S1. Sentence selection distribution.** Frequency of each sentence position among sentences annotated as global extractive summaries by human volunteers.

As can be seen in Figure S1, the closer a sentence is to the beginning of the articles, the more likely it is to be selected as a part of the global summary. Manual inspection confirms that this phenomenon directly reflects the lead bias of news articles in our working data set.
