# Supplementary material for: Targeting COVID-19 and Human Resources for Health News Information Extraction: Algorithm Development and Validation
Source: JMIR AI. 2024 Oct 30;3:e55059. doi: 10.2196/55059 (PMC11561429; doi:10.2196/55059)
Supplement: Multimedia Appendix 3 [file ai_v3i1e55059_app3.docx]

# EIOS classification

Here, we describe the rules-based classification protocol used within EIOS to build the initial data set. This first stage of classification narrows down to news articles relevant to the impact of the COVID-19 pandemic on health workers. Keep news articles which:

1. Include a COVID-19 related term:

   {*coronavirus, covid-19, sars*}
2. **OR** include a keyword among:

   {*health, healthcare, health care, medica*l}

   **AND** a keyword among:

   {*specialist, provider, professional, practitioner, doctor, worker, personnel, staff*}.
3. **OR** include a keyword among:

   {*physician, general practitioner, therapist, nurse, midwife*, caregiver, care provider, gastroenterologist, surgeon, dentist, psychiatrist, pharmacist, ophthalmologist, cardiologist, anaesthesiologist, anesthesiologist, dermatologist, endocrinologist, geriatrician, haematologist, hematologist, nephrologist, neurologist, oncologist, otolaryngologist, pulmonologist, paediatrician, pediatrician, radiologist, urologist*}

Applied to the January 2020 to April 2022 (included) period, such filtering yields 3,235,657 news articles.
