# Supplementary material for: Targeting COVID-19 and Human Resources for Health News Information Extraction: Algorithm Development and Validation
Source: JMIR AI. 2024 Oct 30;3:e55059. doi: 10.2196/55059 (PMC11561429; doi:10.2196/55059)
Supplement: Multimedia Appendix 4 [file ai_v3i1e55059_app4.docx]

# Data set statistics

Here, we give statistics on the initial data set retrieved from EIOS.

The corpus contains 3,235,657 news articles. The corpus made of the body of all news articles has 2,558,783,164 words including 4,782,732 unique words. The corpus made of the title of all news articles contains 42,524,603 words including 391,170 unique ones. The most frequent words in the body and most frequent words in the title (excluding stop words and punctuation) are shown as follows:


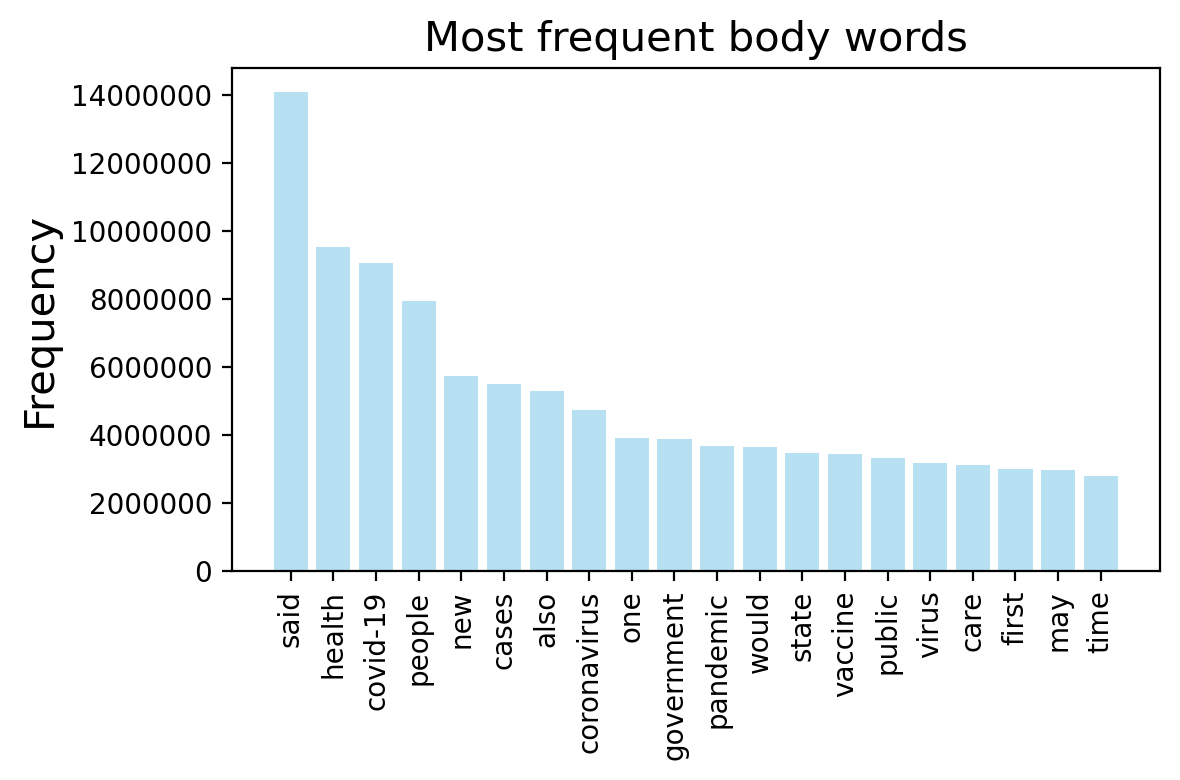

**Figure S2. Most frequent words among articles bodies.**


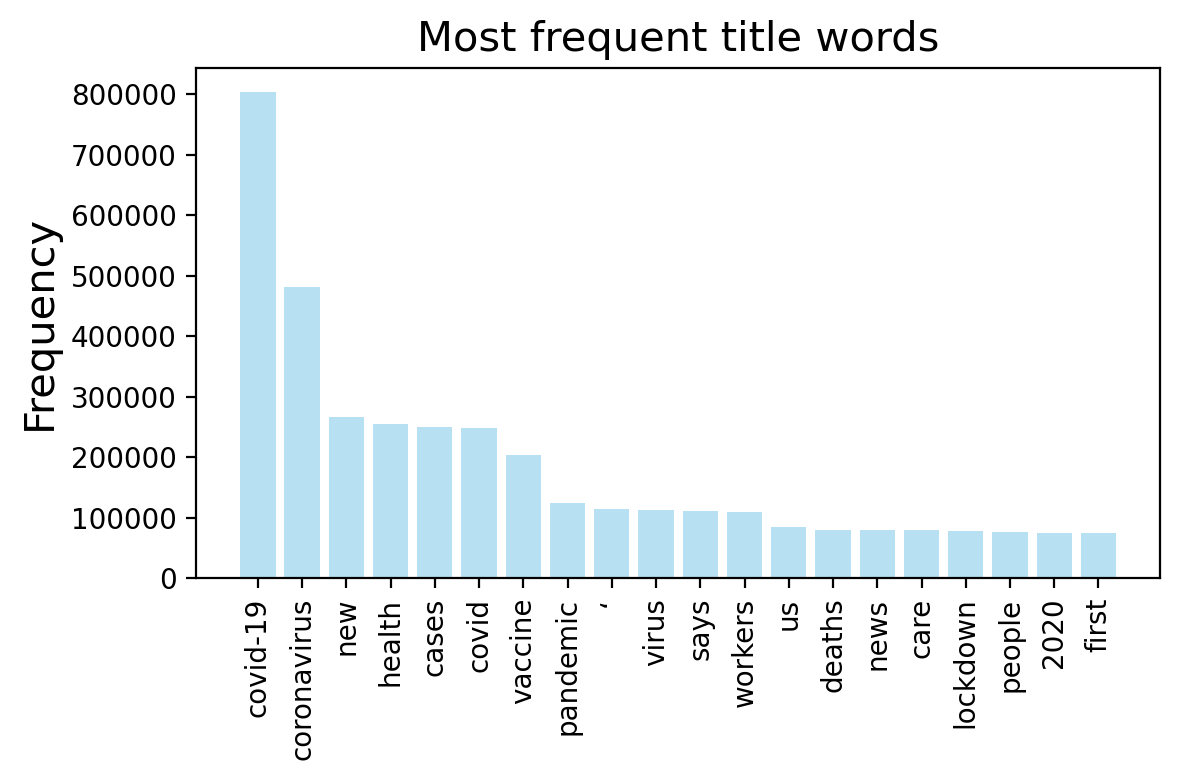

**Figure S3. Most frequent words among articles titles.**

Each article body has on average 791.41 words, and each title has on average 13.15 words. The length distribution of articles body and title is shown as follows:


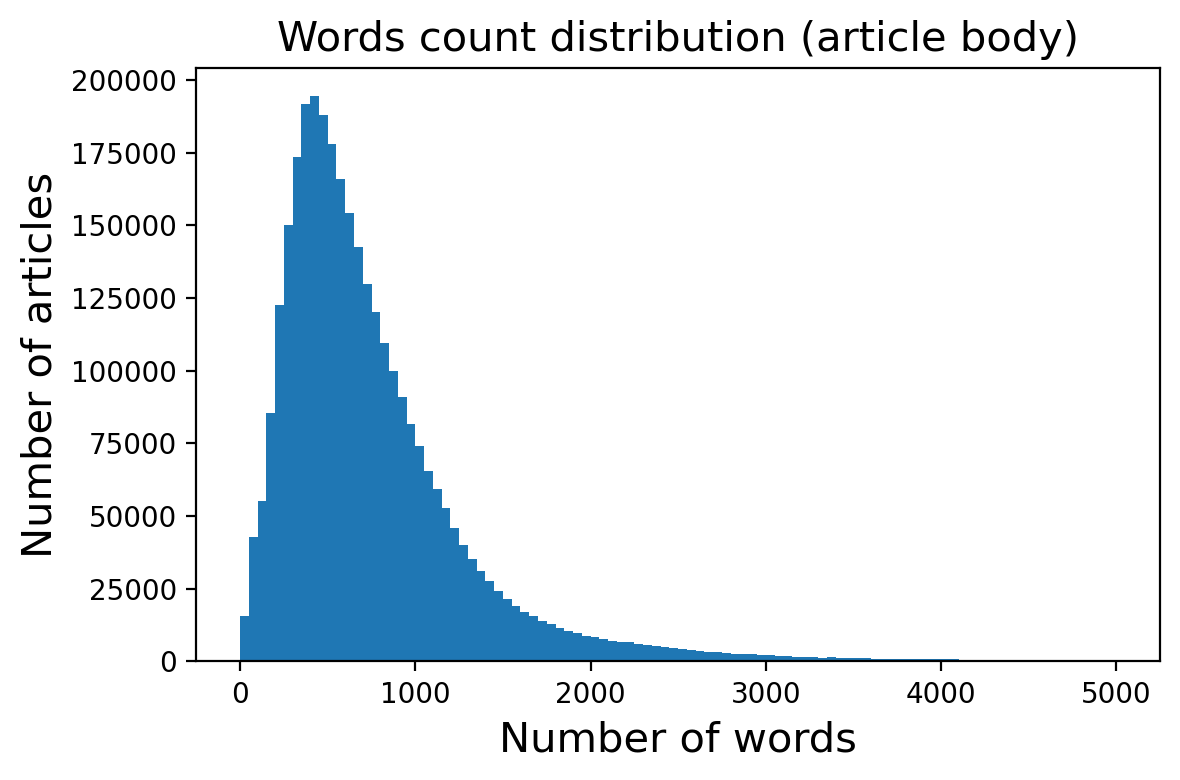

**Figure S4. Length distribution of articles bodies** (in terms of word counts). Articles with more than 5,000 words are not taken into account, to facilitate visualization.


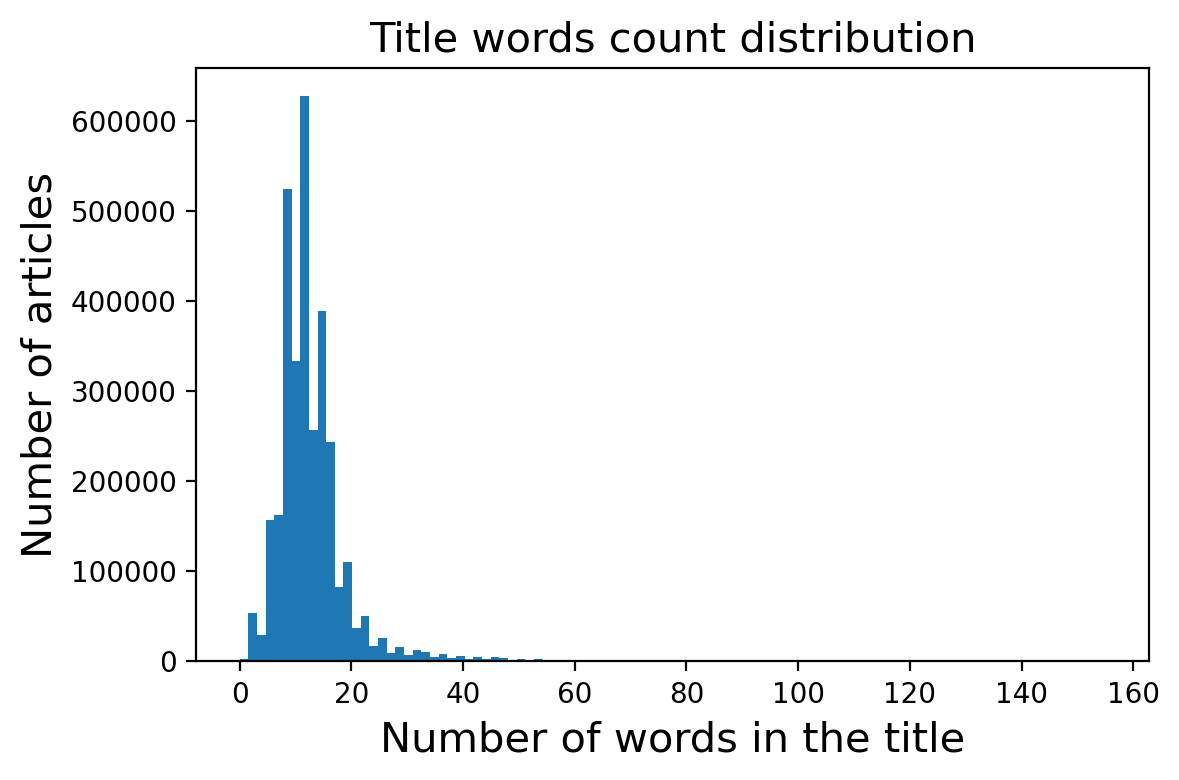

**Figure S5. Length distribution of articles titles** (in terms of word counts).

There are 243 unique location tags, with on average 2.06 different tagged locations per article. The distribution of most frequent locations is shown below:


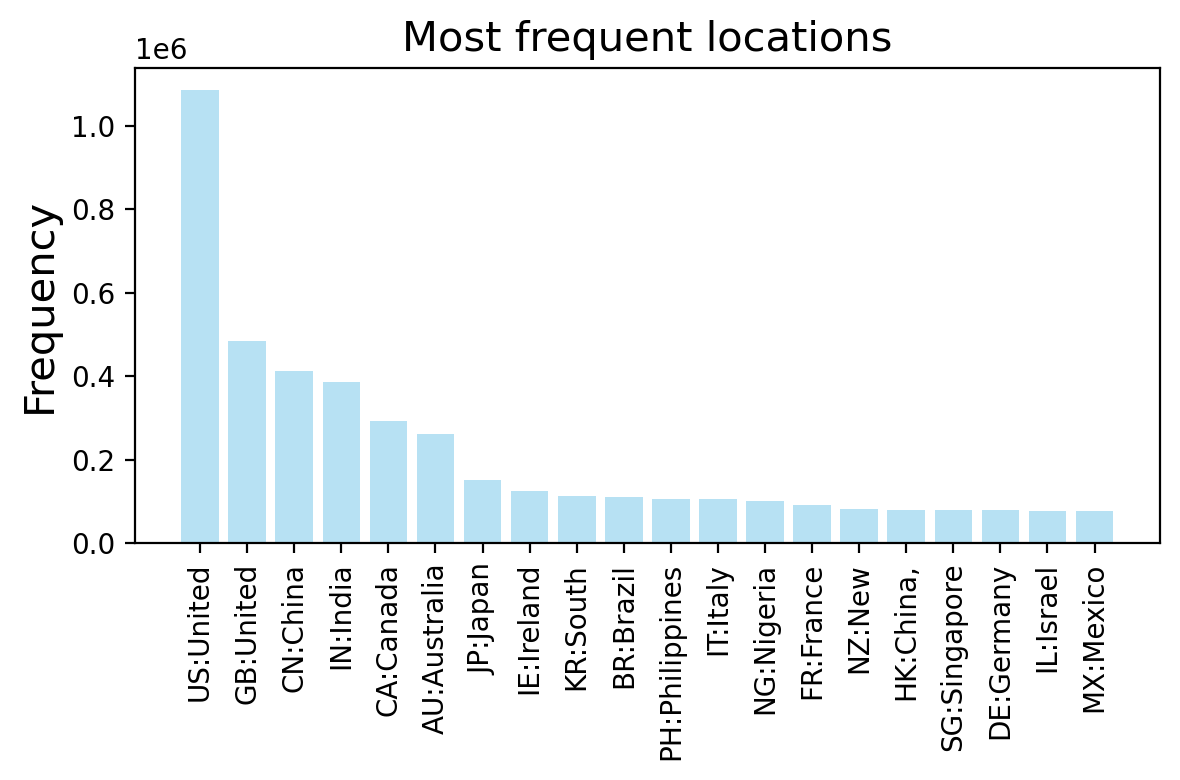

**Figure S6. Top 20 most frequent locations.**

Articles come from a total of 3,472 unique sources, with a unique source per article. Most common sources are:


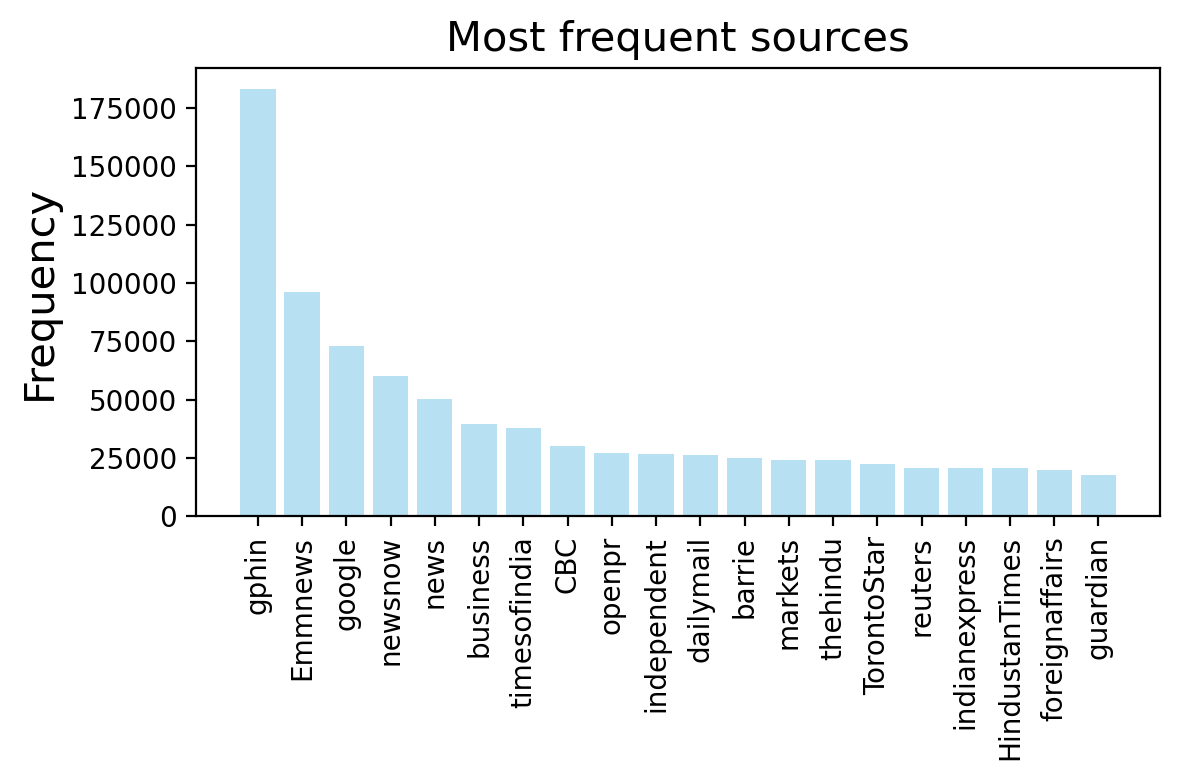

**Figure S7. Top 20 most frequent news sources.**
